# Efficacy and safety of *Eurycoma longifolia* (Physta^®^) water extract plus multivitamins on quality of life, mood and stress: a randomized placebo-controlled and parallel study

**DOI:** 10.29219/fnr.v62.1374

**Published:** 2018-10-16

**Authors:** Annie George, Jay Udani, Nurhayati Zainal Abidin, Ashril Yusof

**Affiliations:** 1 Institute of Biological Sciences, Faculty of Science, University of Malaya, Kuala Lumpur, Malaysia; 2 Biotropics Malaysia Berhad, Lot 21, Jalan U1/19, Section U1, Hicom-Glenmarie Industrial Park, 40150 Shah Alam, Malaysia; 3 Agoura Hills, CA, USA; 4 Exercise Science, Sports Centre, University of Malaya, 50603 Kuala Lumpur, Malaysia

**Keywords:** Eurycoma longifolia, multivitamins, quality of life, mood, stress

## Abstract

**Background:**

The use of alternative and complementary medicines to alleviate stress has increased to avoid the negative effects of pharmaceutical drugs.

**Objective:**

This study investigated the safety and efficacy of *Eurycoma longifolia* in combination with multivitamins (EL+MV) versus placebo on improving quality of life (QoL), mood and stress in moderately stressed healthy participants.

**Methods:**

This randomised, double-blind, placebo-controlled 24-week study enrolled 93 participants aged 25–65 years, with a body mass index of 18–30 kg/m^2^, scoring ≤18 in tension and ≤14 in fatigue subscale of Profiles of Mood Scores (POMS) questionnaire and supplemented with EL+MV or placebo. The primary endpoints were QoL measured by 12-Item Short Form Health Survey (SF-12) questionnaire and mood measured by POMS. The secondary endpoint was stress measured by Multi-Modal Stress Questionnaire (MMSQ). The safety of the intervention product was measured by complete metabolic panel, lipid and renal analysis including several immune parameters.

**Results:**

While there were no significant between-group differences, within-group improvements were observed in the SF-12 QoL, POMS and MMSQ domains. In the SF-12 domain, improvements were seen in role limitation due to emotional health (*P* = 0.05), mental component domain (*P* < 0.001), emotional well-being (*P* < 0.001), social functioning (*P* = 0.002) as well as vitality (*P* = 0.001) at week 12. An increasing trend in POMS-vigour domain was also observed in the EL+MV group at week 12. A 15% decrease in physical stress domain (*P* < 0.05) compared with 0.7% in the placebo group was also observed in MMSQ. When the subjects were subgrouped according to age, 25–45 and 46–65 years of age, for primary outcomes, between-group significance was observed in the 25–45 year group in the social functioning domain of SF-12 (*P* = 0.021) and POMS-vigour (*P* = 0.036) in the 46–65 year group. No significant changes were observed in vital signs and complete metabolic panel. Regarding immune parameters, the lymphocytes increased significantly in the active group (*P*≤0.05). In total, 13 adverse events were reported: six on placebo and seven on EL+MV.

**Conclusion:**

EL+MV may support the QoL, mood, stress and immune parameters in healthy participants.

**Trial registration:**

This study has been registered at clinicaltrials.gov (NCT02865863).

Unresolved stress greatly increases the risk of developing depression, consequently becoming a topic of public health awareness and therapeutic interventions. Depression afflicts approximately 20–25% of women and 10–17% of men during their lifetime (1). While certain drugs like fluoxetine (Prozac) and sertraline (Zoloft) have been used to treat stress and anxiety disorders and, in recent years, the anti-depressant setraline, there is a concern that one can be addicted to and dependent on drug usage (2). Alternative and complementary medicines, such as herbal supplements, have emerged as substitutes to conventional therapeutics for ameliorating depression and maintaining mental well-being (3–5).

In South East Asia, where traditional/herbal medicine is popular, supplementation with *Eurycoma longifolia* Jack, Simaroubaceae (*Tongkat Ali* or Malaysian ginseng), has been shown to be efficacious for alleviating stress (6), as well as many other ailments including fever, arthritis, high blood pressure, diabetes, low energy or libido, bacterial infections and cancer (7–9). *Eurycoma longifolia* (EL) is a slender evergreen tree mainly found in Malaysia, Indonesia and the Philippines. Derivatives of this plant have been used to restore and enhance energy levels, to improve physical and mental performance, endurance and stamina (9) and quality of life (QoL), as evidenced by a decrease in aging males symptoms score and an increase in serum testosterone levels (10). Another related study showed improvement in QoL and sexual well-being in men, specifically in the domains of ‘physical function’ and ‘vitality’ (11). The roots of EL are largely responsible for its biological activity due to the presence of alkaloids, quassinoids, quassinoid diterpenoids, eurycomacoside, eurycolactone, laurylcolactone or eurycomalactone and pasakbumin-B (7) and peptides (12). It has been demonstrated to reduce stress through the reduction of cortisol (6) with a concurrent increase in lymphocytes and natural killer cells (13). These active ingredients in EL and in other plants, such as mountain ginseng (*Panax ginseng*), may be responsible for improving QoL, as well as combating stress without adverse effects (11, 14–16). A recent 4-week, randomised clinical study on moderately stressed participants consuming water extract from EL reported significant improvements in mood, tension, anger and confusion (6). This was accompanied by a reduction in cortisol and increased testosterone levels. Another study investigated the effect of EL on the immune status of moderately stressed subjects (13), whereby a 1-month supplementation of 200 mg EL extract per day nearly significantly improved vigour measured by Profile of Mood States (POMS) while scores for immunological vigour also improved.

Micronutrient deficiencies contribute to stress and depression (5). Indeed, low levels of folic acid may be correlated with depressive symptomatology (16) that can be ameliorated by mineral supplementation (17). Multinutrient formulations have a significantly greater effect in reducing stress and anxiety in subjects than single interventions alone (18). In addition, a recent study demonstrated that a formulation consisting of multivitamins, minerals and herbal extracts was more effective than placebo in significantly reducing the overall score on a depression, anxiety and stress scale, as well as improving alertness and general daily functioning in healthy older men (19). Furthermore, a high dose of vitamin B complex with vitamin C and minerals led to significant improvements in ratings on Perceived Stress Scales, General Health Questionnaire and the ‘vigour’ subscale of POMS in healthy males (20).

While preclinical and clinical studies lend credence to the ability of EL to improve mood which was possibly linked to hormonal balance favouring elevated mood (21), efficacy studies of EL in combination with micronutrients and conducted in accordance with established standards are currently lacking. The objective of this study was to investigate the safety and efficacy of a multivitamin mix in combination with EL water extract on QoL, mood and stress of moderately stressed but healthy participants.

## Materials and methods

This study was conducted in accordance with the Guidelines for Good Clinical Practice (ICH-6) and the Declaration of Helsinki. Institutional Review Board (IRB) approval was obtained from IntegReview Ethical Review Board, an independent IRB located in Austin, TX, USA, comprising scientific and non-scientific members of mostly medical doctors, on 17 January 2014 prior to initiation of any study-related activities. The IRB reviewed the protocol, medical ethics, informed consent, advertisement, stipend and compliance to protocol. The study was conducted at Medicus Research LLC, a clinical research site located at Agoura Hills, CA, USA. Written informed consent was obtained from volunteers prior to all study procedures. The recruitment and follow-up took place from 7 February 2014 to 13 March 2015.

### Study design

This was a randomised, double-blind, placebo-controlled parallel study with a 12-week efficacy and a 24-week safety period. The allocation *ratio* of participants in each of the comparison groups was 1:1. Efficacy was measured at 6 and 12 weeks, with safety and adverse events at 6, 12 and 24 weeks. The participants were required to make a total of four visits to the clinical trial site at Medicus Research LLC, Agoura Hills, CA.

At screening/baseline (week 0), inclusion/exclusion criteria, medical history and concomitant therapies were reviewed; baseline demographic data were collected; heart rate, respiratory rate, blood pressure and oral temperature were measured; and body mass index (BMI) was calculated. Fasting blood samples were obtained for assessment of complete blood count (CBC), comprehensive metabolic panel (CMP) including kidney function (estimated glomerular filtration rate, blood urine nitrogen [BUN], creatinine and bilirubin), liver function (aspartate aminotransferase, alanine transaminase), lipid panel (total cholesterol [TC], high-density lipoprotein-cholesterol [HDL-C], low-density lipoprotein-cholesterol [LDL-C], and triglycerides), testosterone (free and total) and urinalysis (leukocyte esterase, amorphous and calcium oxalate crystals). A urine pregnancy test was conducted on females with child-bearing potential. Electrocardiogram (EKG) was performed and POMS (22), SF-12 QoL (23), and Multi-Modal Stress Questionnaire (MMSQ) (24) were administered. Participants were dispensed a 6-week supply of the investigational product, a daily dosing diary and a 3-day food recall. Subjects who met all the study inclusion criteria and none of the exclusion criteria were enrolled in the study. After eligibility was confirmed, all volunteers received a randomisation number.

Participants returned to the clinic at weeks 6, 12 and 24 after having fasted for 10 h for assessment of medical and concomitant medication history. Vital signs and anthropometric measures, compliance and adverse events, and current medical history were reviewed. Fasting blood was collected for CBC, CMP, lipid panel, testosterone (free and total) measurements and urinalysis was performed. EKG was performed at baseline and at week 24.

POMS, SF-12 and MMSQ questionnaires were administered and a daily dosing diary and a 3-day food recall were dispensed at baseline, week 6 and week 12 only. At week 6, participants were dispensed a 6-week supply of the investigational product and at week 12, a 12-week supply of the investigational product. Participants maintained their daily diary for the duration of the study period and were required to record concomitant therapies and adverse events.

### Participants

Study participants were recruited from the general population by online advertising, recruiting and available clinical trial databases. Inclusion criteria were as follows: healthy volunteers between 25 and 65 years of age, BMI 18–30 kg/m^2^ and having self-reported moderate stress. Moderate stress was defined as a measure of both the tension and fatigue subscale of the POMS questionnaire. Participants who scored ≤18 in the tension subscale and ≤14 in the fatigue subscale were considered as having moderate stress. The tension subscale items are tense, on edge, uneasy, restless, nervous and helpless. A highest score (4 = extremely) for each of these items will give a total subscale score of ≤24 in tension and ≤20 in fatigue. An upper cut-off limit was determined, that is, a scoring of ≤18 in the tension subscale and ≤14 in the fatigue subscale, to exclude subjects who might fall within extremely stressed and possibly depressed category that will require medication and possibly cannot be addressed with health supplementation of multinutrients. The subjects were furthermore required to answer a Yes/No questionnaire in the inclusion/exclusion criteria as to whether they perceived themselves to having mid-level stress at work as a result of employment and life balance.

Exclusion criteria: participants were excluded if they were pregnant, lactating, planning to become pregnant or unwilling to use adequate contraception during the duration of the study, or had a history of immune system disorders, neurological disorders, temporal arthritis, ulcerative colitis, history of cancer within 2 years prior to enrolment, any active infection, or infection requiring antibiotics within 30 days of enrolment, significant gastrointestinal conditions including, but not limited to, inflammatory bowel disease, eating disorders, untreated hypothyroidism and use of herbal products containing androgenic/anxiolytic activity within 30 days prior to enrolment.

### Investigational product

The investigational product (50 mg per tablet) was a proprietary water extract of EL root (Physta^®^ also known as LJ100 in the USA). The multivitamin mix consisted of ascorbic acid (50 mg), retinyl acetate (4,000 IU), cholecalciferol (200 IU), Dl-alpha tocopherol acetate (15 IU), thiamine mononitrate (1.5 mg), riboflavin (1.7 mg), pyridoxine hydrochloride (2 mg), cyanocobalamin (0.001 mg), folic acid (0.2 mg), niacinamide (20 mg), D-biotin (0.15 mg), copper (2 mg), iron (10 mg), magnesium (10 mg), manganese (2.5 mg), selenium (0.005 mg), zinc (5 mg) and calcium (100 mg). The placebo contained microcrystalline cellulose, polyvinylpyrrolidone, sodium starch glycolate, colloidal silicon dioxide and magnesium stearate. The investigational product was produced under good manufacturing practices (GMP) requirements by Unison Nutraceutical Sdn Bhd and stored in a dry place at room temperature. Participants were instructed to consume either EL+MV or the placebo starting the day following the baseline visit, one tablet daily in the morning with water for 24 weeks.

### Outcome measures

The primary and secondary outcomes measures were assessed by questionnaires at week 0 (Visit 0), week 6 (Visit 1) and 12 (Visit 2). The primary outcome measure for this study was the assessment of the efficacy of EL+MV versus placebo on mood and QoL. Mood state was assessed using the POMS questionnaire, which consisted of the following: total mood disturbance and its subscales, tension, depression, anger, fatigue, confusion and vigour. The POMS rated emotional and physical aspects of mood as ranging from ‘not at all (1 point)’ to ‘extremely (5 points)’. A lower score, except for vigour, indicates better mood.

The POMS Iceberg profile, designed for assessing active/healthy individuals (25), was also analysed. QoL was assessed by the SF-12 questionnaire which measured the following domains: physical component summary, mental component summary, physical functioning, role limitations due to physical health, role limitations due to emotional health, energy/fatigue ratio (vitality), emotional well-being, social functioning, pain and general health. Scores on the SF-12 scales ranged from 0 to 100, with higher scores indicating better health.

The secondary objective was to assess stress using MMSQ, which measured the following subscales: total, behavioural, cognitive and physical. The MMSQ rated emotional and physical aspects of stress as ranging from ‘never (1 point)’ to ‘constantly (5 points)’.

Safety and tolerability of the investigational product were assessed through changes in CBC, CMP, lipid panel, total and free testosterone, urinalysis and vital signs at all visits. The study would be temporarily stopped for any of the following: if WHO Grade 3 toxicity is experienced by four or more patients or WHO Grade 4 toxicity is experienced by two or more patient(s).

### Compliance

The dispensed study product compliance diaries were returned to the clinic and participants who were non-compliant with their diaries were reminded of their obligations regarding appropriate study compliance.

### Sample size

The sample size was calculated using the G*Power 3.0.10 software based on a reference value proposed by Perazzo et al. (26), which assessed QoL (SF-12) following treatment of Gerovital (a multivitamin and mineral combined with Panax ginseng extract) and placebo. In addition, a between-factor repeated-measure analysis of variance (ANOVA) with a level of significance (*α*) of 0.05 (two sided) and power of 80% was considered, while the ratio between trial and control group was set at 1, resulting in 36 subjects per group. A 20% loss to follow-up was considered relevant, thus resulting in 45 subjects per group.

### Randomisation

Stratified randomisation sequences were created with computer-generated random numbers, which allocated subjects based on sex (male/female) into two groups. Demographic stratifications based on gender were set and crossed. Patients were randomly assigned to order of treatment (placebo or active) using simple randomisation based on the atmospheric noise method and sequential assignment was used to determine group allocation (GraphPad Prism 6). A computer-generated list of random numbers was used in order to allocate participants. The results of these two randomisations were combined and assigned as the final randomisation sequence for this trial. Allocation, enrolment and assignment of participants to products were performed by the staff of Medicus Research LLC who did not perform any analyses or clinical procedures. The allocation information was disclosed to the investigator, subjects and a statistician after all measurements were completed. The investigational product was stored in a sequentially numbered Study Product Container in a locked cabinet with limited access.

### Statistical analysis

The modified per-protocol analysis included subjects with at least one post-dose completed visit and participants who completed all visits of the 24-week study and consumed the product. Subgroup analysis was performed based on gender for testosterone measurements, domains within questionnaires and age groups. The safety analysis was based on all randomised participants known to have taken at least one dose of the investigational or placebo products. Subgroup analysis of primary endpoints was performed for the age groups, 25–45 and 46–65 years, due to perceived stress from increased responsibilities in the older age group and potential hormonal variances which affect QoL in these subgroups.

All evaluations were performed using the software package R 3.2.2 (R Core Team, 2015). Descriptive statistics were calculated for each group and statistical comparisons were performed using the analysis of covariance (ANCOVA) adjusting for baseline values. Numerical endpoints that are intractably non-normal were assessed by the Mann-Whitney U test; in these instances, only the comparisons of the changes from baseline were considered in the formal testing between groups. Statistical comparisons for baseline characteristics, lipid and testosterone levels, and measures of safety (haematology, blood chemistry, anthropometrics and vital signs) were performed using ANOVA. For categorical endpoints, the differences in proportions between groups were formally tested by the Fisher’s exact test. The Shapiro–Wilk normality test was carried out to determine data normality when *P* > 0.05. Within-group comparisons on numeric endpoints were made using Student’s paired *t*-test or, in the case of intractable non-normality, the Wilcoxon signed rank test. Differences were considered significant at *P* ≤ 0.05. Subgroup analysis based on gender differences was conducted for testosterone measurements only.

## Results

### Participant baseline characteristics

**Fig. 1:**
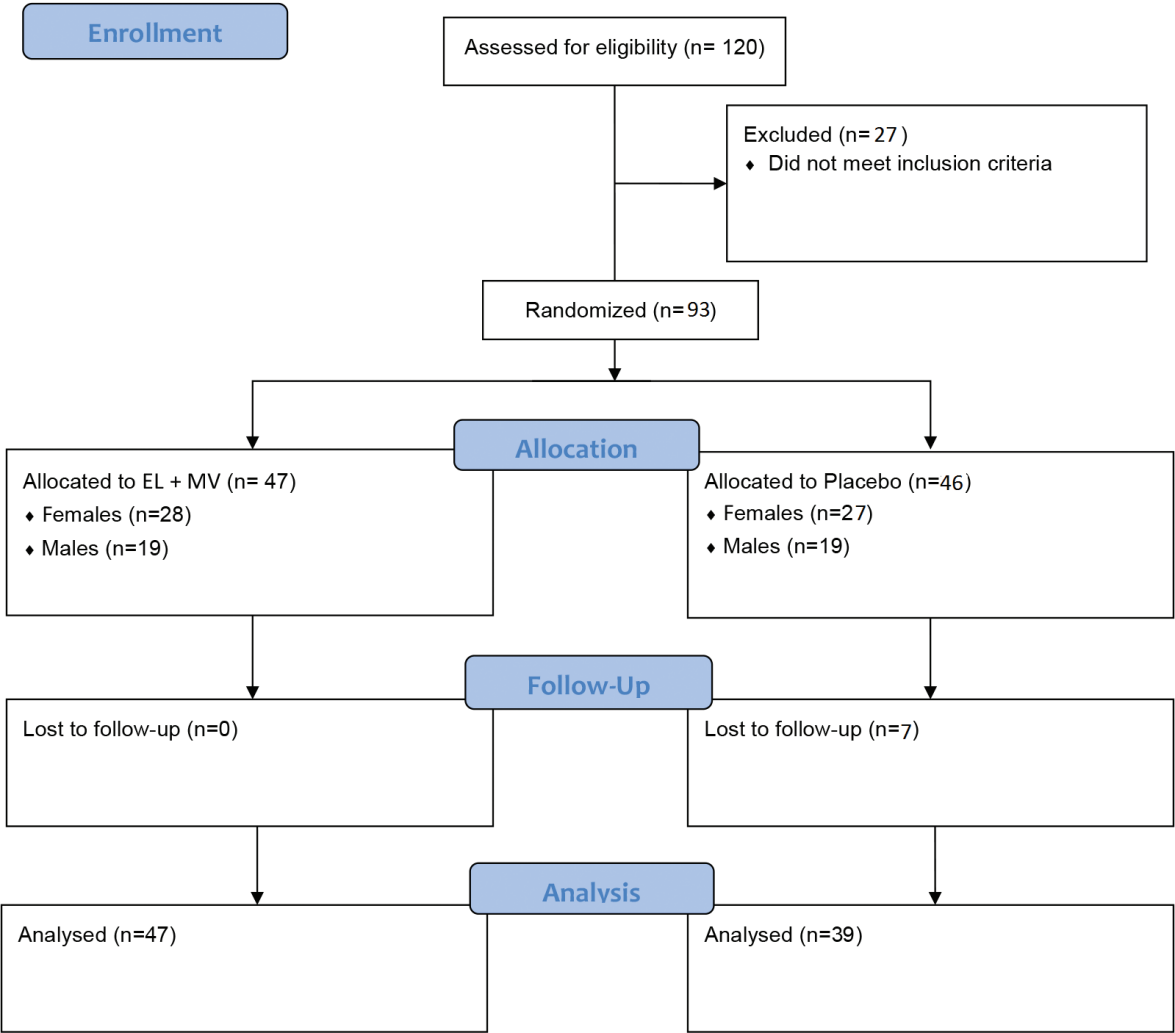
Study flowchart. A total of 120 participants were screened, of which 93 were randomised in the study, with 47 in the EL+MV group and 39 in the placebo group enrolledas the modified per-protocol population in the final analysis. EL+MV, *E. longifolia* + multivitamin.

A total of 120 participants were screened and a total of 93 subjects were enrolled, of which 7 were lost to follow-up due to the long enrolment period of 6 months (Fig. 1). There were 28 females and 19 males in the EL + multivitamins group (EL+MV), and 20 females and 19 males in the placebo group. The demographic characteristics of participants were not significantly different in terms of age, BMI, employment and relationship status between groups at baseline (Table 1).

**Table 1:** Demographics and anthropometric measures of all 86 participants enrolled in the study.

	EL+MV (*n* = 47)	Placebo (*n* = 36)	*P*-value§
Age (years) Mean ± SD Median (min – max)	40.6 ± 12.3 36 (25 – 62)	41.0 ± 9.9 39.5 (25 – 62)	0.744σ
Gender (*n*[%]) Female Male	28 (60%) 19 (40%)	18 (50%) 18 (50%)	0.504
BMI (kg/m^2^) Mean ± SD (*n*) Median (min – max)	23.9 ± 3.20 23.4 (18.1 – 35)	25.0 ± 3.24 24.8 (18.3 – 31.8)	0.124
Tobacco use (*n*[%]) Current smoker Non-smoker Past smoker	7 (16%) 31 (69%) 7 (16%)	4 (11%) 29 (81%) 3 (8%)	0.505
Alcohol use (*n*[%]) Current consumer Non-drinker Past drinker	31 (67%) 11 (24%) 4 (9%)	22 (61%) 12 (33%) 2 (6%)	0.739
Ethnicity (*n*[%]) African-American Asian Caucasian Latino/Hispanic Other	5 (11%) 3 (6%) 32 (68%) 3 (6%) 4 (9%)	4 (11%) 2 (6%) 18 (51%) 7 (20%) 4 (11%)	0.384
Current employment (*n*[%]) Employed Not employed	44 (96%) 2 (4%)	33 (94%) 2 (6%)	1.000
Relationship status (*n*[%]) Divorced Domestic partnership Married Separated Single Widowed	4 (9%) 1 (2%) 12 (26%) 0 (0%) 28 (60%) 2 (4%)	3 (8%) 1 (3%) 8 (22%) 4 (11%) 19 (53%) 1 (3%)	0.318
Have children (*n*[%]) No Yes	32 (71%) 13 (29%)	18 (50%) 18 (50%)	0.067
Systolic blood pressure (mmHg) Mean ± SD (*n*) Median (min – max)	117.7 ± 14.3 117 (91 – 154)	118.4 ± 13.5 119 (91 – 150)	0.819
Diastolic blood pressure (mmHg) Mean ± SD (*n*) Median (min – max)	74.5 ± 10.6 73 (50 – 105)	77.0 ± 10.9 75 (60 – 99)	0.296
Heart Rate (beats per minute) Mean ± SD (*n*) Median (min – max)	66.2 ± 10.7 66 (42 – 94)	64.5 ± 9.5 65 (41 – 84)	0.468
Body temperature (°F) Mean ± SD (*n*) Median (min – max)	98.14 ± 0.44 98.2 (97.2 – 99.8)	98.16 ± 0.64 98.1 (96.4 – 99.6)	0.868
Respiratory rate (per minute) Mean ± SD (*n*) Median (min – max)	14.57 ± 1.96 15 (12 – 20)	14.95 ± 1.60 15 (12 – 18)	0.354

σ Between-group comparison was made using the independent Student’s *t*-test.

§ Between-group comparisons were performed using Fisher’s exact test. The variable *n* indicates the number of subjects analysed. Demographics data were not available for three participants.

More than 94% of participants in both groups were employed and were predominantly Caucasian in ethnicity. There were no significant differences in CBC, CMP and urinalysis, anthropometric measures and vital signs between groups at baseline (Table 2). Analysis of POMS-Tension-Anxiety mood state subscale showed participants to be moderately stressed.

**Table 2:** CBC and CMP safety parameters assessed in participants at all visits.

Item	Reference value	Group	Screening (Week 0)	Week 6	Week 12	Week 24
AST	7 –≤70 U/L	EL + MV	24.8 ± 10.1	23.9 ± 7.8	29.3 ± 27.1	21.5 ± 7.9
		Placebo	25.2 ± 9.0	23.7 ± 10.2	24.3 ± 11.1	29.1 ± 36.1
ALT	12 – <90 U/L	EL + MV	21.5 ± 13.4	21.5 ± 9.4	22.9 ± 14.5	21.2 ± 16.7*
		Placebo	23.5 ± 14.9	21.1 ± 12.4*#	22.0 ± 13.5**	22.3 ± 16.2
ALP	39−117 IU/L	EL + MV	64.5 ± 19.2	63.7 ± 19.8	63.8 ± 19.1	59.2 ± 20.3
		Placebo	66.9 ± 18.9	67.1 ± 18.4	67.5 ± 17.1	66.1 ± 19.7
Total bilirubin	≤25 μmol/L	EL + MV	0.62 ± 0.36	0.53 ± 0.32	0.454 ± 0.284	0.463 ± 0.213
		Placebo	0.62 ± 0.36	0.53 ± 0.33	0.450 ± 0.244	0.474 ± 0.350
Sodium	133–148 mmol/L	EL + MV	141.18 ± 1.85	141.30 ± 2.47	140.8 ± 4.7	138.9 ± 6.5
		Placebo	141.17 ± 2.50	141.08 ± 2.20	140.8 ± 4.9	141.0 ± 2.8
Potassium	3.3–5.7 mmol/L	EL + MV	4.21 ± 0.35	4.39 ± 1.25	4.24 ± 0.37	4.20 ± 0.37
		Placebo	4.17 ± 0.27	4.24 ± 0.32	4.61 ± 1.74*	4.36 ± 0.39*
Chloride	98–115 mmol/L	EL + MV	102.48 ± 1.97	102.42 ± 2.29	101.5 ± 3.9	99.2 ± 5.7***
		Placebo	103.17 ± 2.66	102.73 ± 1.79	101.9 ± 4.1*	100.9 ± 2.8***
Carbon dioxide	18–29 mmol/L	EL + MV	26.98 ± 1.99	28.6 ± 11.3	25.4 ± 3.4*	23.43 ± 2.93***
		Placebo	26.64 ± 2.26	24.9 ± 3.9*	24.8 ± 2.8*	23.97 ± 2.65***
Anion gap	3–11 mEq/L	EL + MV	12.3 ± 3.5	13.3 ± 5.0	16.6 ± 5.6***	20.0 ± 3.3***
		Placebo	11.7 ± 3.2	14.8 ± 5.7*	17.0 ± 5.3***	19.8 ± 4.2***
Calcium	8.4–10.4 mg/dL	EL + MV	9.61 ± 0.40	9.60 ± 0.40	9.26 ± 1.58	9.04 ± 0.67***
		Placebo	9.56 ± 0.41	9.51 ± 0.41	9.47 ± 0.59	9.19 ± 0.45**
Glucose	70–109 mg/dL	EL + MV	94.9 ± 8.6	93.5 ± 9.6	95.2 ± 11.4	89.0 ± 11.6**
		Placebo	92.2 ± 10.2	93.3 ± 6.4	91.6 ± 10.5	88.5 ± 16.3
Blood urea nitrogen	8.0–20.0 mg/dL	EL + MV	13.2 ± 3.1	13.4 ± 4.4	12.8 ± 3.1	14.7 ± 11.3
		Placebo	13.4 ± 3.7	12.9 ± 4.0	12.9 ± 5.2	13.1 ± 4.1
Creatinine	0.47–0.79 mg/dL	EL + MV	0.795 ± 0.152	0.782 ± 0.147	0.777 ± 0.172	0.770 ± 0.204
		Placebo	0.794 ± 0.200	0.768 ± 0.220	0.812 ± 0.247	0.774 ± 0.192
Blood urea nitrogen: creatinine ratio	10:1 –20:1	EL + MV	17.1 ± 4.7	17.8 ± 5.3	17.1 ± 4.2	17.8 ± 4.8
		Placebo	17.6 ± 5.3	17.6 ± 5.9	16.3 ± 5.0	17.4 ± 4.9
Estimated glomerular filtration rate	50–≥120 mL/min/1.73 m^2^	EL + MV	64.6 ± 13.1	69.2 ± 19.5	78.2 ± 23.2***	92.1 ± 28.2***
		Placebo	62.6 ± 9.5	74.9 ± 32.4**#	83.5 ± 30.9**	93.1 ± 23.6***
Total serum protein	6.7–8.3 g/dL	EL + MV	7.13 ± 0.37	7.09 ± 0.41	8.0 ± 6.2	6.72 ± 0.56***#
		Placebo	7.13 ± 0.47	7.02 ± 0.45	7.0 ± 0.6	7.09 ± 0.48
Serum albumin	3.5 to 5.5 g/dL	EL + MV	4.63 ± 0.29	4.60 ± 0.30	4.59 ± 0.43	4.42 ± 0.48**#
		Placebo	4.54 ± 0.34	4.56 ± 0.33	4.56 ± 0.38	4.55 ± 0.31
Globulin	2.6–4.6 g/dL	EL + MV	2.493 ± 0.277	2.49 ± 0.30	2.36 ± 0.29*	2.31 ± 0.51***#
		Placebo	2.586 ± 0.304	2.46 ± 0.35*	2.45 ± 0.33	2.53 ± 0.32
Albumin : globulin ratio	0.8–2.0	EL + MV	1.877 ± 0.251	1.875 ± 0.260	1.958 ± 0.205	1.99 ± 0.35*
		Placebo	1.778 ± 0.251	1.897 ± 0.320*#	1.894 ± 0.269*	1.83 ± 0.30
Total cholesterol	120–219 mg/dL	EL + MV	195 ± 46	194 ± 43	192 ± 43	180 ± 36*
		Placebo	184 ± 34	184 ± 41	184 ± 40	177 ± 40
Triglycerides	30–149 mg/dL	EL + MV	89 ± 52	104 ± 99	111 ± 90	91 ± 54
		Placebo	98 ± 75	111 ± 171	118 ± 110	97 ± 53
HDL cholesterol	40–95 mg/dL	EL + MV	74.8 ± 22.6	73.7 ± 23.2	72.7 ± 24.9	61.5 ± 17.9***
		Placebo	69.3 ± 20.5	67.5 ± 24.3	63.0 ± 20.9**	61.3 ± 18.7&**
LDL cholesterol	65–139 mg/dL	EL + MV	102 ± 38	100 ± 41	99 ± 34	103 ± 33
		Placebo	95 ± 28	95 ± 29	97 ± 31	99 ± 28
Coronary risk factor (cholesterol : HDL)	<3.3	EL + MV	2.79 ± 0.99	2.87 ± 1.07	4.3 ± 8.6*	3.26 ± 1.18***
		Placebo	2.87 ± 1.03	3.07 ± 1.71	3.1 ± 1.1**	3.15 ± 1.06**
VLDL cholesterol	2 to 30 mg/dL	EL + MV	17.9 ± 10.4	20.9 ± 19.7	22.1 ± 18.1	18.2 ± 10.9
		Placebo	19.5 ± 14.9	22.3 ± 34.3	23.6 ± 22.0	19.4 ± 10.6
White blood cell	3,300–9,000/μL	EL + MV	6.06 ± 1.65	5.60 ± 1.55*	5.57 ± 1.47*	5.88 ± 1.41
		Placebo	5.93 ± 1.86	6.10 ± 2.07	5.86 ± 1.58	5.43 ± 1.34
Red blood cell	430–570 10^4/mL	EL + MV	4.71 ± 0.44	4.69 ± 0.49	4.64 ± 0.42*	4.63 ± 0.45*
		Placebo	4.79 ± 0.47	4.70 ± 0.46	4.70 ± 0.42	4.60 ± 0.40***
Haemoglobin	M: 13.5–17.5 g/dL	EL + MV	14.69 ± 1.29	14.58 ± 1.44	14.38 ± 1.32**	14.30 ± 1.42**
	F: 11.5–15.0 g/dL	Placebo	14.61 ± 1.45	14.40 ± 1.49	14.44 ± 1.53	13.98 ± 1.36***
Haematocrit	M: 39.7–52.4%	EL + MV	43.2 ± 3.5	43.3 ± 3.7	43.1 ± 3.4	43.0 ± 3.7
	F: 34.8–45.0%	Placebo	43.3 ± 3.6	42.8 ± 3.3	43.4 ± 3.8	42.3 ± 3.4*
Blood platelet	14.0–34.0 ×10^3/mm^3^	EL + MV	248 ± 57	255 ± 61#	256 ± 80	277 ± 76**
		Placebo	227 ± 57	223 ± 50	239 ± 49*	245 ± 81*
Mean corpuscular volume	85–102 fl	EL + MV	92.1 ± 3.9	92.6 ± 4.4	92.9 ± 4.3*	93.0 ± 4.1*
		Placebo	90.7 ± 5.4	91.4 ± 5.2	92.3 ± 4.9*	90.7 ± 11.6
Mean corpuscular haemoglobin	28.0–34.0 pg	EL + MV	31.22 ± 1.38	31.11 ± 1.47	31.00 ± 1.50	30.91 ± 1.14*
		Placebo	30.57 ± 2.13	30.67 ± 2.13	30.72 ± 2.10	30.42 ± 2.02*
Mean corpuscular haemoglobin concentration	30.2–35.1%	EL + MV	33.95 ± 0.92	33.61 ± 1.12	33.39 ± 1.22*	33.24 ± 0.99***
		Placebo	33.74 ± 1.15	33.57 ± 1.43	33.26 ± 1.24*	33.01 ± 1.19***
Neutrophil count	1.6–8.0 ×10^9/L	EL + MV	55.5 ± 10.8	53.5 ± 10.9#	52.2 ± 10.6*	54.9 ± 9.5
		Placebo	56.1 ± 10.1	57.3 ± 9.8	55.8 ± 9.0	56.8 ± 7.7
Lymphocyte count	0.8–3.0 ×10^9/L	EL + MV	32.5 ± 9.2	34.6 ± 9.8	35.4 ± 9.1*#	33.2 ± 8.4
		Placebo	32.6 ± 8.6	32.4 ± 9.5	32.4 ± 6.9	32.9 ± 6.6
Monocyte count	0.1–1.5 × 10^9/L	EL + MV	7.33 ± 2.01	8.00 ± 2.32	7.93 ± 1.94	8.09 ± 2.47
		Placebo	7.42 ± 1.67	7.27 ± 1.90	8.19 ± 2.72	7.65 ± 1.91
Eosinophil count	0.0–0.7× 10^9/L	EL + MV	3.2 ± 4.0	3.08 ± 2.20	3.28 ± 2.47	3.22 ± 2.35
		Placebo	2.8 ± 3.3	2.40 ± 1.93	2.45 ± 1.97	2.25 ± 1.20
Basophil count	0.0–0.2× 10^9/L	EL + MV	1.56 ± 0.89	1.42 ± 1.32	1.23 ± 1.76**	0.60 ± 0.54***
		Placebo	1.29 ± 0.56	1.08 ± 0.59	1.17 ± 2.30**	0.48 ± 0.46***

* *P* < 0.05,

** *P* < 0.01,

*** *P* < 0.001, significant within-group differences and

# significant between-group differences in *E. longifolia* + multivitamins (EL+MV) group (*n* = 44–47) and the placebo group (*n* = 34–36).

### Primary endpoints

#### SF-12 questionnaire on QoL

POMS, SF-12 and MMSQ scores obtained by all participants in the study before and after supplementation with EL+MV group or placebo are presented in Table 3. There were no significant between-group differences reported in physical component, mental component, physical functioning, role limitations due to physical health, role limitations due to emotional health, vitality, emotional well-being, social functioning, pain and general health domains as assessed by the SF-12 questionnaire, but several within-group significant findings were observed (Table 3).

**Table 3:** POMS and SF-12 MMSQ scores in all participants in the study.

	Before supplementation		*P*-value	After supplementation–week 6		*P*-value	After supplementation – week 12		*P*-value
	EL+MV	Placebo		EL+MV	Placebo		EL+MV	Placebo	
**POMS†**									
Tension-anxiety	8.3 ± 4.4	9.8 ± 5.7	0.289	6.6 ± 4.0**	6.8 ± 4.2***	0.909	5.4 ± 2.9 ***	6.4 ± 4.0***	0.489
Depression-dejection	8.7 ± 9.5	10.6 ± 11.5	0.892	5.7 ± 9.1**	5.6 ± 8.0**	0.821	3.5 ± 5.4***	5.2 ± 7.7*	0.427
Anger-hostility	5.1 ± 5.1	7.2 ± 8.5	0.682	3.9 ± 6.3	4.5 ± 6.3*	0.976	2.4 ± 3.5**	4.2 ± 5.8	0.162
Vigour-activity	15.0 ± 6.1	16.6 ± 6.1	0.237	15.1 ± 6.6	16.4 ± 6.1	0.453	16.3 ± 5.4	16.5 ± 5.8	0.974
Fatigue-inertia	6.2 ± 5.7	6.7 ± 5.5	0.673	5.2 ± 5.4	5.5 ± 4.7	0.634	4.2 ± 4.6*	3.2 ± 3.9***	0.268
Confusion-bewilderment	6.0 ± 4.0	7.9 ± 4.9	0.108	4.96 ± 2.88	5.19 ± 2.72**	0.521	4.63 ± 2.03	5.31 ± 3.01*	0.576
Overall mood	19 ± 27	25 ± 36	0.651	11.2 ± 27.3*	11.1 ± 25.3**	0.962	3.8 ± 18.6***	7.9 ± 23.8**	0.485
**SF-12**†									
Physical component	56.2 ± 5.5	54.9 ± 4.8	0.294	54.1 ± 5.8*	55.1 ± 3.2	0.332	53.8 ± 4.6**	53.7 ± 5.3	0.930
Mental component	28.4 ± 11.2	32.0 ± 10.8	0.148	33.4 ± 9.9**	35.8 ± 8.6**	0.266	35.4 ± 9.4***	36.0 ± 8.9	0.749
Physical functioning	53.4 ± 7.6	52.5 ± 9.4	0.781	52.7 ± 9.3	54.7 ± 5.4	0.413	53.7 ± 6.3	54.0 ± 7.7	0.391
Role limitations-physical	28.43 ± 2.60	28.88 ± 1.98	0.444	28.84 ± 1.93	29.14 ± 1.31	0.517	28.74 ± 2.01	28.62 ± 2.18	0.817
Role limitations-emotional	18.5 ± 4.7	20.5 ± 3.9	0.044	20.3 ± 3.6**	20.9 ± 3.5	0.341	20.2 ± 4.2*	21.6 ± 2.9	0.101
Energy/fatigue	44.7 ± 11.6	48.3 ± 11.7	0.158	47.3 ± 10.6	51.2 ± 10.0	0.081	49.7 ± 10.1**	51.2 ± 10.6	0.465
Emotional well-being	34.6 ± 13.0	40.7 ± 11.6	0.274	37.5 ± 12.7**	42.8 ± 11.7*	0.429	42.7 ± 10.8***	43.5 ± 11.6**	0.797
Social functioning	46.7 ± 9.9	49.1 ± 8.9	0.284	49.5 ± 8.5	52.8 ± 6.5*	0.066	52.0 ± 6.3**	50.2 ± 8.2	0.446
Pain	54.3 ± 5.2	54.2 ± 5.4	0.966	53.2 ± 7.6	55.4 ± 4.8	0.207	53.0 ± 7.0	54.5 ± 5.3	0.404
General health	56.6 ± 5.6	56.1 ± 5.1	0.487	56.5 ± 5.5	56.1 ± 6.0	0.843	55.9 ± 5.2	54.7 ± 7.9	0.888
**MMSQ**†									
Physical stress	47.6 ± 13.6	43.8 ± 13.0	0.198	45.2 ± 13.1	43.5 ± 11.2*	0.071	40.4 ± 9.1*	40.3 ± 9.5	0.277
Behavioural stress	22.1 ± 5.5	21.6 ± 5.0	0.700	20.6 ± 5.2*	19.6 ± 3.4**	0.323	19.6 ± 4.7***	19.2 ± 4.5***	0.673
Cognitive stress	16.5 ± 6.0	14.2 ± 5.8	0.094	14.0 ± 5.4***	12.2 ± 3.9**	0.091	12.4 ± 3.7***	11.8 ± 4.2**	0.520
Overall stress	86.1 ± 23.0	79.7 ± 21.3	0.209	79.8 ± 21.4*	72.2 ± 14.6**	0.064	75.5 ± 18.0***	71.3 ± 15.7**	0.181

POMS, SF-12 and MMSQ scores are depicted as mean ± SD.

* *P* ≤ 0.05,

** *P* ≤ 0.01,

*** *P* ≤ 0.001, significant within-group difference in *E. longifolia* + multivitamins (EL+MV) (*n* = 47) and placebo group (*n* = 36).

† Between-group comparisons were made using ANCOVA.

**Fig. 2:**
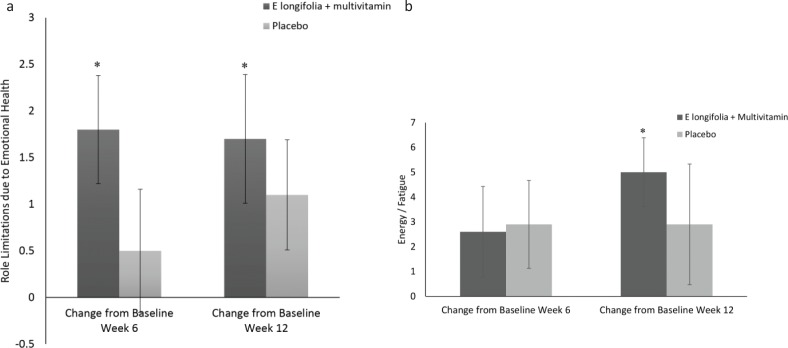
(a) Changes in role limitation due to emotional health in EL+MV (*n* = 46) and placebo (*n* = 35) groups. Participants consuming *E. longifolia* + multivitamins displayed a significant improvement in role limitation due to emotional health at week 6 (9.7%; *P* = 0.003) and week 12 (9.2%; *P* = 0.05) when compared to baseline. Axes represent change in scores that numerically capture domains in the SF-12 questionnaire. Within-group comparisons were made using the paired *t*-test. Mean ± SE values. *P ≤ 0.05. (b) Energy/fatigue ratio in EL+MV (*n* = 46) and placebo (*n* = 35) groups. Only participants consuming *E. longifolia* + multivitamins showed a significant increase (11.2%, *P* = 0.001) in their energy/fatigue ratio at week 12 when compared to baseline. Axes represent change in scores that numerically capture domains in the SF-12 questionnaire. Within-group comparisons were made using the paired *t*-test. Mean ± SE values. *P ≤ 0.05.

Participants supplemented with EL+MV reported significant improvements from baseline, with 9.7% improvement in role limitation due to emotional health at week 6 (*P* = 0.003) and 9.2% at week 12 (*P* = 0.05) (Fig. 2a) and a further 11.3% improvement in vitality (energy/fatigue ratio) at week 12 (*P* = 0.001) (Fig. 2b). Similar improvements from baseline were not reported by participants in the placebo group.

Participants supplemented with EL+MV reported a significant increase in the mental component domain at weeks 6 (*P* = 0.001) and 12 (*P* < 0.001), with an increase of 24.6% in the EL+MV compared to 12.7% in the placebo group at week 12. The placebo group only had significant improvements at week 6 (*P* = 0.007). In the emotional well-being domain, significant improvements were observed in both groups, with a 23% (*P* < 0.001) and 6.9% (*P* < 0.01) improvement observed at week 12, respectively, in the EL+MV and placebo groups. The social functioning domain for participants supplemented with EL+MV significantly improved by 11.3% at week 12 (*P* = 0.002) but only by 7.5% at week 6 (*P* = 0.01) in the placebo group.

A subgroup analysis of subjects based on age group 25–45 years had *n* = 29 on treatment and *n* = 24 on placebo, while age subgroup 46–65 years had *n* = 18 on treatment and *n* = 12 on placebo. Primary and secondary outcome measures of POMS, SF-12 and MMSQ revealed a 14.4% increase in the social functioning score within the SF-12 questionnaire in the 25–45-year subgroup of the EL+MV group, achieving a between-group significance (*P* = 0.021). Changes in other domains remained non-significant.

#### Profile of mood states questionnaire on mood

There were no significant between-group differences reported in total mood disturbance, tension, depression, anger, fatigue, confusion and vigour assessed by POMS questionnaire. Within group, participants supplemented with EL+MV and placebo reported significant improvements in several of the POMS domains. An increasing trend was observed in the vigour domain of the EL+MV group at week 12.

**Fig. 3:**
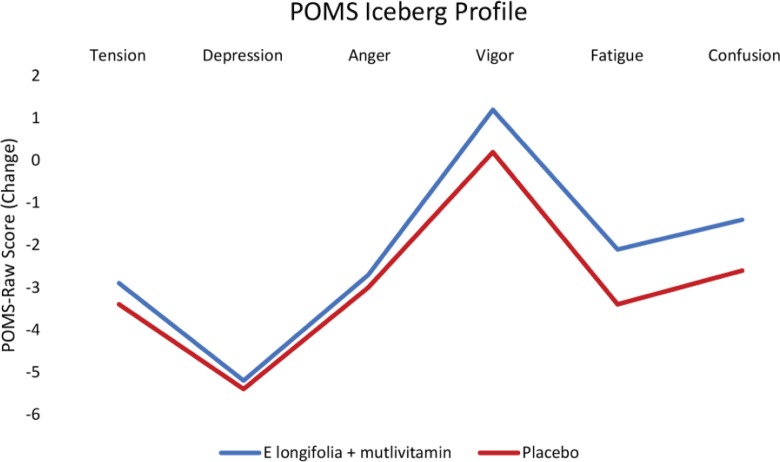
Changes in POMS Iceberg profiles in EL+MV (*n* = 46) and placebo (*n* = 36) groups. Changes in POMS Iceberg profiles based on the raw POMS mood state subscales were consistent with that of healthy and active individuals in tension, depression, anger, vigour, fatigue and confusion in *E. longifolia* + multivitamins and placebo groups. Vigour activity in placebo group was reduced compared to an increase in the supplemented group. Axes represent change in scores that numerically capture domains in POMS Iceberg profile.

The POMS Iceberg profile was applied to the POMS raw scores of healthy, moderately stressed population of participants in this study. Average baseline profiles showed that participants in both groups had the expected normal profiles. A normal profile consists of a peak in vigour with tension, depression, anger, fatigue and confusion making up the trough of the profile (Fig. 3). In the subgroup analysis of the POMS scores, participants between the ages of 46 and 65 years showed significant between-group improvement in vigour (*P* = 0.036) by 14.1% in the EL+MV group, observed by the mean change from weeks 0 to 12.

### Secondary outcomes

#### Multi-modal stress questionnaire on stress

There were no significant between-group differences in self-reported total, behavioural, cognitive and physical stress by participants, as assessed by the MMSQ questionnaire (Table 3).

Significant within-group effects were observed in several domains in both groups, but only for the EL+MV group, significant reduction in physical stress was observed at week 12 (*P* < 0.05), as evidenced by a reduction of 15% compared to 0.7% in the placebo group only at week 6 (*P* < 0.05). The decrease in cognitive stress and total stress in the EL+MV group was significant (*P* < 0.001) compared to the placebo group (*P* < 0.01) at week 12.

#### Compliance

Compliance, which was assessed by counting the returned unused test product at each visit, was calculated by determining the number of dosage units taken divided by the number of dosages expected to have been taken multiplied by 100. The overall mean compliance was greater than 99% in both EL+MV and placebo groups. No participants were removed from the study due to low compliance (less than 80%).

#### Safety parameters

Anthropometric measures and vital signs (systolic and diastolic blood pressure, body temperature, respiratory rate and heart rate) were similar between EL+MV and placebo groups after 24 weeks of supplementation. Participants consuming EL+MV showed incidental differences in their respiratory rate at week 12 (*P* = 0.03) and mean diastolic blood pressure at week 6 (*P* = 0.02) compared to the placebo, but not at other time points (Table 1). However, all excursions were within a normal clinical reference range for the duration of the study.

Reduction in neutrophil count at week 6 (*P* = 0.03) and an increase in lymphocyte count at week 12 (*P* = 0.01) versus placebo were observed (Table 2). Mean platelet volume increased in the EL+MV at weeks 12 (*P* < 0.001) and 24 (*P* < 0.001) and in the placebo group at weeks 6 (*P* = 0.03), 12 (*P* < 0.001) and 24 (*P* < 0.001) compared to baseline, but all values remained within their normal laboratory range (Table 2).

Participants in the EL+MV group showed a decrease in glucose concentration (*P* = 0.005) and TC(*P* = 0.03) at 24 weeks compared to baseline (Table 2). Urinalysis revealed a difference in the presence of leukocyte esterase at week 6 (*P* = 0.008), with 25% of participants in the EL+MV group testing negative (Table 4). Nine per cent more participants in the placebo group tested positive for the presence of calcium oxalate crystals in the urine compared to EL+MV group (Table 4).

**Table 4:** Urinalysis of all participants in the study based on the number of subjects (*n*).

Presence of leukocyte esterase (n)					Presence of calcium oxalate crystals (n)			
		EL+MV	Placebo	*P*-value§		EL+MV	Placebo	*P*-value§
Week 0 (screening)	1+ 2+ 3+ Negative Trace	5 (11%) 2 (4%) 1 (2%) 34 (74%) 4 (9%)	1 (3%) 0 (0%) 1 (3%) 32 (94%) 0 (0%)	0.121	Few None	0 (0%) 44 (100%)	3 (9%) 31 (91%)	0.079
Week 6	1+ 2+ 3+ Negative Trace	2 (5%) 0 (0%) 2 (5%) 38 (88%) 1 (2%)	0 (0%) 2 (6%) 0 (0%) 25 (76%) 6 (18%)	0.008	Few None	3 (9%) 31 (91%)	7 (27%) 19 (73%)	0.085
Week 12	1+ 2+ 3+ Negative Trace	3 (7%) 1 (2%) 1 (2%) 35 (85%) 1 (2%)	2 (6%) 1 (3%) 1 (3%) 21 (66%) 7 (22%)	0.108	Few None	1 (6%) 17 (94%)	2 (17%) 10 (83%)	0.548
Week 24	1+ 2+ 3+ Negative Trace	2 (5%) 3 (7%) 0 (0%) 35 (80%) 4 (9%)	0 (0%) 2 (6%) 0 (0%) 28 (88%) 2 (6%)	0.744	Few None	2 (25%) 6 (75%)	2 (50%) 2 (50%)	0.547

§ Between-group analysis was made using the Fisher’s exact test. *P*≤0.05 is statistically significant.

#### Testosterone levels

**Fig. 4:**
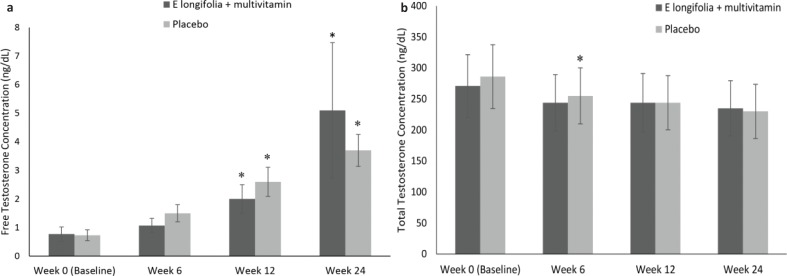
(a) Serum total testosterone levels in EL+MV (*n* = 44) and placebo groups (*n* = 36). Serum total testosterone decreased significantly (*P* = 0.009) in the placebo group at week 6 compared to baseline. Within-group comparisons were made using the paired *t*-test. Mean ± SE values.*P ≤ 0.05. (b) Serum free testosterone levels in EL+MV (*n* = 44) and placebo (*n* = 36) groups. Serum free testosterone increased significantly in both groups (*P* < 0.001). Larger percentage increases were observed in the *E. longifolia* + multivitamins group. Within-group comparisons were made using the paired *t*-test. Mean ± SE values.*P ≤ 0.05.

There was no between-group significance; however, a significant time effect within group changes in testosterone levels was observed. Serum total testosterone decreased in the placebo group at week 6 compared to during screening (*P* = 0.009) (Fig. 4a). This decrease continued till weeks 12 and 24, but was not observed in the EL+MV group. In contrast, free serum testosterone levels increased in both groups (*P* < 0.001) (Fig.4b).

However, there was an increase in free testosterone levels in males supplemented with EL+MV from 1.67 ± 2.35 ng/dL at baseline to 11.4 ± 24.9 ng/dL, double the increase seen in the placebo group, from 1.4 ± 12.7 ng/dL to 6.4 ± 2.9 ng/dL (Table 5). An increase in free testosterone levels from 0.15 ± 0.3 ng/dL in the EL+MV and 0.12 ± 0.4 ng/dL in the placebo group at baseline to 1.05 ± 0.9 ng/dL and 1.3 ± 1.3 ng/dL, respectively, at week 24 was observed in female participants. There were no significant between-group differences in free testosterone levels in both genders.

**Table 5:** Mean concentrations of testosterone in female and male participants.

	EL+MV	Placebo	*P*-value (*t*-test)†	EL+MV	Placebo	*P*-value (*t*-test)†
	Female			Male		
	Mean ± SD (*n*) Within Group *P*-value	Mean ± SD (*n*) Within Group *P*-value		Mean ± SD (*n*) Within Group *P*-value	Mean ± SD (*n*) Within Group *P*-value	
**Total testosterone concentration (ng/dL)**						
Week 0 (baseline)	15.9 ± 12.7 (26)	20.5 ± 12.6 (19)	0.270	641 ± 198 (18)	582 ± 177 (17)	0.269
Week 6	18.3 ± 14.2 (26)	17.8 ± 9.6 (20)	0.723	570 ± 193 (18)	468 ± 230 (17)	0.184
	21.6 ± 22.0 (23)	29.4 ± 28.6 (17)	0.155	563 ± 189 (16)	458 ± 188 (17)	0.118
Week 24	18.6 ± 13.2 (28)	20.0 ± 13.1 (19)	0.737	573 ± 208 (18)	434 ± 154 (16)	0.070
Change from weeks 0 to 6	1.9 ± 6.5 (24) *P* = 0.223‡	−2.9 ± 10.2 (19) *P* = 0.556‡	0.271	−64 ± 125 (17) *P* = 0.058‡	−124 ± 156 (15) *P* = 0.003‡	0.246
Change from weeks 0 to 12	5.3 ± 14.0 (21) *P* = 0.203‡	8.6 ± 33.3 (16) *P* = 0.660‡	0.530	−74 ± 153 (16) *P* = 0.094‡	−130 ± 152 (15) *P* = 0.005‡	0.401
Change from weeks 0 to 24	1.9 ± 8.1 (26) *P* = 0.115‡	± 8.5 (18) *P* = 0.433‡	0.839	−79 ± 155 (17) *P* = 0.049‡	−127 ± 185 (14) *P* = 0.017‡	0.710
**Free testosterone concentration (ng/dL)**						
Week-0 (baseline)	0.15 ± 0.30 (26)	0.12 ± 0.44 (19)	0.239	1.67 ± 2.35 (18)	1.40 ± 1.27 (17)	0.298
Week-6	0.39 ± 0.62 (26)	0.27 ± 0.50 (20)	0.456	2.04 ± 2.19 (18)	2.95 ± 2.53 (17)	0.276
Week-12	0.85 ± 0.78 (23)	0.68 ± 1.04 (17)	0.175	3.7 ± 4.2 (16)	4.6 ± 3.1 (17)	0.231
Week-24	1.05 ± 0.89 (28)	1.36 ± 1.37 (19)	0.931	11.4 ± 24.9 (18)	6.4 ± 2.9 (16)	0.506
Change from week-0 to -6	0.16 ± 0.43 (24) *P* = 0.132‡	0.09 ± 0.31 (19) *P* = 0.888‡	0.304	−0.04 ± 1.72 (17) *P* = 0.678‡	1.72 ± 2.51 (15) *P* = 0.030‡	0.086
Change from week-0 to 12	0.62 ± 0.76 (21) *P* < 0.001‡	0.53 ± 0.99 (16) *P* = 0.025‡	0.249	2.0 ± 4.4 (16) *P* = 0.130‡	3.4 ± 3.0 (15) *P* = 0.002‡	0.151
Change from week-0 to-24	0.86 ± 0.93 (26) *P* < 0.001‡	1.26 ± 1.34 (18) *P* < 0.001‡	0.519	9.7 ± 25.9 (17) *P* = 0.005‡	5.3 ± 2.9 (14) *P* < 0.001‡	0.218

‡ Within-group analysis was made using the Wilcoxon signed rank test. Significant within-group difference in *E. longifolia* + multivitamins (EL+MV) and placebo groups;

† Between-group analysis was made using the *t*-test. Probability values *P* ≤ 0.05 are statistically significant; *n* = number of subjects.

#### Adverse events

In this clinical study, there were a total of 13 adverse events reported by 13 participants: six (urinary tract infection, blood in urine, nasal congestion, nasopharyngitis [*n* = 2] and migraine) of which were in the placebo group and 7 (food poisoning, kidney infection, fracture, influenza, vomiting, nausea and urinary tract infection) were in the EL+MV group.

## Discussion

This study evaluated the efficacy and safety of EL+MV in healthy males and females with moderate stress. The demographics of the population studied were middle class and lower middle class individuals who worked hard to sustain their families and maintain their lifestyles while juggling work-related requirements. The participants were employed and experienced self-reported job-related stress due to work responsibilities, particularly when responsibility and authority were mismatched (27).

Participants on EL+MV reported a significant improvement in their mental component domain, suggesting they felt ‘calm and peaceful’, emotional well-being and improvement in energy/fatigue profile after the 12-week supplementation. This supports the results of the POMS analysis with regard to the vigour activity domain, which reported an increasing trend in the EL+MV group. These results were further supported by the POMS Iceberg profiles that showed optimal peaks of vigour activity and a decrease in the negative mood clusters, contributing to the trough values of the profile in both EL+MV and placebo groups. This concept has previously been applied to assess physical activity and mood among healthy individuals (28, 29). After the 12-week supplementation, the POMS Iceberg profiles favoured an improvement in vigour among participants in the EL+MV group. Previous studies with nutritionally enriched coffee (28) and adaptation to competitive sports (30) have reported a similar shift to healthy POMS Iceberg profiles, akin to positive mood states associated with the use of multivitamins and protein supplements in other stressed populations (31). In another study, a significant improvement in mood by a reduction in tension and anxiety domain of the POMS was found (*P* = 0.054) in stressed subjects with EL supplementation (13).

Participants on EL+MV reported significant improvement in role limitation due to emotional health and in social functioning domains, suggesting an enhancement in their QoL, social interactions and related activities. A significant between-group improvement in the 25–45 years subgroup in the social functioning domain could be explained by the higher occurrence of mood and anxiety disorder generally increasing with age (30), hence the extract at a low dosage of 50 mg EL/day, not showing an intervention effect in the older subgroup, instead having an effect in the younger subgroup. In another study, EL with a dosage of 200 mg/day was reported to improve the QoL demonstrated by a reduction of 38% in aging males score (QoL) after 1-month supplementation (10). A higher dosage of EL therefore may be required to affect an older and otherwise healthy population.

The results of this study indicate that the consumption of EL+MV formulation affected the emotional health (SF-12) and vigour (POMS) of the participants. Significant between-group differences favouring the EL group in the vigour activity domain of POMS for the 46–65 years age group could be due to the physical fitness since the reduction in muscle strength in the upper and lower limbs, changes in body fat percentages and endurance increase with age and poor nutrition (31). Hence, an intervention effect may have probably arisen from muscle and strength improvement (32, 33), anti-ageing and enhancement of vigour (13) properties of EL. In addition, participants consuming EL+MV showed a significant decrease in glucose concentrations from baseline to the end of the study, supporting its previously reported anti-hyperglycaemic properties *in vivo* (34, 35) which overall may contribute to well-being of subjects.

Improvement in mood with the highest decrease in cognitive stress subscale in the MMSQ – which is made up of several questions that include a participant’s perception of ‘feeling out of control’, ‘inability to concentrate’, ‘feeling no good’ and a general sense of things being ‘really bad’ and a desire to ‘run away and hide’ – in participants supplemented with EL+MV is possibly due to the previously reported calming effect of EL (6), which is corroborated by animal studies demonstrating the anti-anxiolytic effects of EL (22). It was observed that there were more parents with children in the placebo group. It is surprising however that the mean for MMSQ (stress) at baseline was lower in all four domains in the placebo group in spite of them having more children. The POMS, however, had higher baseline means in individual domains. The SF-12 had mixed baseline values where either placebo or treatment group had higher baseline values. There were no between-group differences in all domains at baseline. There were also more women in the treatment group, which may have contributed to higher mean at baseline in MMSQ compared to placebo since stress was more prevalent among women (36).

A large placebo effect as well as large standard deviations in POMS total mood disturbance and its subscales, however, perhaps contributed to the absence of between-group significance in the questionnaires tested. Furthermore, it is also plausible that the lower dose of EL (50 mg/day) used in the current formulation may not have provided the clinical benefits achieved with the higher dose (200 mg/day) used in previous studies which showed improvements in tension, anger and confusion with EL supplementation (6). A reduction in negative mood states mediated by phytochemicals has been demonstrated in numerous studies, with placebo effects ranging from 1 to 50% (37–41); therefore, the 10–12% placebo effect seen with SF-12 and MMSQ and nearly 70% in POMS in the current study is not surprising. Thus, the lower dose of EL and a substantial placebo effect exacerbated by the large statistical deviations observed in the current study may have obscured the efficacy of EL+MV. This is a challenge in clinical trials conducted on a healthy population as the effects of nutrition interventions are subtle, whereas drug trials compare exposure with no exposure, and nutrition trials compare higher and lower exposures. Everyone consumes nutrients in their diet; therefore, subtle differences may be difficult to detect and have long latency periods. Taken together, these limitations and considerations mean that it is difficult to demonstrate statistically significant benefit between groups (42). In addition, due to the lack of significant difference between groups in primary and secondary outcomes, a comparison was made in both outcomes across groups and within groups at multiple time points, and also in subgroup analyses by sex and age. This could contribute to type II error, lack of between-group statistical significance and false positives. The problem of multiple comparisons to be counteracted by, for example, Bonferroni analysis, may be considered.

Within the safety parameters, significant increase in lymphocytes similar to earlier reports (15, 43, 44) was observed. Furthermore, micronutrients contribute to the body’s natural defences by supporting physical barriers (skin/mucosa), cellular immunity and antibody production. Vitamins A, B6, B12, C, D, E and folic acid and the trace elements iron, zinc, copper and selenium work in synergy to support the protective activities of the immune cells, whereby vitamins A, C, E and zinc assist in enhancing the skin barrier function (45). Combining EL with micronutrients thus is anticipated to provide health benefits through hormonal balance and optimal nutritional requirements.

Participants in this study showed a significant decrease in neutrophils that degranulate to release proteases during pathogenesis and psychological stress (46). Stress also enhances neutrophilia and neutrophil counts (47) without concurrent increase in eosinophils or monocytes (48), which was also noted in this study. Plant extracts are known to reduce leukocyte esterase (49) and calcium oxalate crystals (50) in urine, similar to observations made in this study, which suggests fewer urinary abnormalities associated with EL+MV. It can be speculated that EL is a nutritional adaptogen (51), an agent that rejuvenates the body through restoration, which may regulate neutrophils and leukocyte esterase release. It is plausible that the EL+MV-mediated improvement in emotional health and vitality may be associated with changes in these immune parameters.

Importantly, serum total testosterone levels in the EL+MV group did not alter, while it decreased in the placebo group. The stress hormone cortisol increases under stressed states and as a result, the opposite effect is that the testosterone levels dip. It is possible through the absence of the hormone modulating effect of EL and multinutrients, the cortisol levels as a result of stress may have increased, hence causing the reduction in testosterone levels (52). However, there was an increase in free testosterone levels in males in the EL+MV group. Increase in free testosterone levels is a measure of bioavailable testosterone (53). Our results are in agreement with other studies showing a 10.3% increase in free testosterone with EL in combination of *Polygonum minus* supplementation compared to 4.3% with the placebo (54), and EL-mediated enhancement of free testosterone levels by 46.8% in subjects suffering from hypogonadism (10). A supplementation with testosterone improves mood, energy, friendliness and decreased negative mood (55). Eurypeptides, a bioactive peptide of 4.3 kDa with testosterone-modulating properties identified in EL (10), may restore normal testosterone levels by influencing the release of free testosterone from its binding hormone, sex-hormone-binding globulin, which results in improvement in QoL (10, 55). Eurypeptides enhance metabolism of pregnenolone and progesterone to yield more dehydro-epiandrosterone and androstenedione (10, 44, 56) by activating the CYP17 (17α-hydroxylase and 17,20lyase) enzyme (10). In addition, even though levels of free testosterone increased significantly from baseline in females in both groups, the increase was higher in the placebo group compared to EL+MV group, rendering it non-significant between groups. Therefore, EL+MV and the adaptogenic nature of EL may be considered safe in women, preventing an increase in free testosterone, which is related to conditions such as hirsutism and polycystic ovary syndrome (57).

There were no significant and sustained changes from baseline or against placebo in relevant blood, liver and kidney laboratory tests. This product was well-tolerated and safe in the population studied, with no serious adverse events reported, which corroborates findings from previous randomised and controlled clinical trials evaluating EL (11). This study did not measure cortisol levels, which perhaps may have provided valuable information to understand the efficacy of EL+MV on various stress indicators and immunological parameters. This is a limitation of the study and should be considered when conducting future clinical studies.

Observational studies and clinical trials evaluating the efficacy of EL on mood, stress and testosterone levels have consistently shown favourable changes in these parameters, thereby providing a rationale for its incorporation into new formulations of multivitamins. Multivitamin supplementation enhanced mood by 15% and energy levels by 17% (58) and reduced depressive symptoms since inadequacy of key micronutrients has been associated with poor mood states (19). Therefore, it is reasonable to speculate that EL synergises health benefits exerted by multivitamins through improvement in mood states, vigour and a reduction in stress. The effect of intervention on depressed subjects could be evaluated in the future since the subjects used in this study were healthy subjects with only mid-level stress and not in a depressed state. There are differences and similarities in the way drugs affect a depressed mental state compared to the product. For example, fluoxetine (Prozac) and sertraline (Zoloft) are newer medicines that act as selective serotonin reuptake inhibitors (SSRIs). The product in this study appears to affect energy and mood levels most likely via hormonal modulation (testosterone) and nutritional supplementation, for example, vitamins B complex and C, which also affect mood (20). Vitamin B complex is involved in the metabolism of S-adenosylmethionine (SAM), a donator of methyl groups, which plays a decisive role in the functioning of the nervous system and in the formation of neurotransmitters (e.g. serotonin) (59). The target of the vitamins is similar, whereas the target of EL is different for this study. There could be a lack of intervention effect in subjects with chronic stress or depressed state; hence, one needs to be open to a more prescription-based therapy than nutritional supplementation for beyond everyday moderate stress. With unrealistic expectations to treat depression or stress related to suffering from, for example, advanced disease, there is a risk of dropping traditional medication exacerbated with a fear of potential interactions between EL and other medications. It is however noteworthy that recent research on herb–drug interaction of EL was weak and inconclusive due to the dissimilarities between investigated solvent extract and aqueous extract of EL (60).

## Conclusions

This study reports significant within-group improvements in QoL, mood and stress of moderately stressed participants supplemented with EL+MV for 12 weeks. Despite the placebo effects, participants supplemented with EL+MV reported improvements in vigour, mental component, emotional well-being, cognition and testosterone levels possibly through hormonal balance and nutritional supplementation. The stress-related changes in neutrophils and leukocyte esterase suggest the counteracting effect of EL+MV supplementation; hence, further research is warranted. Significant between-group improvements in the social functioning domain of SF-12 observed in the 25–45 years age group and vigour domain of POMS in the 46–65 years age group supplemented with EL+MV indicate the efficacy of the supplement in particular spheres of influence, particularly relating to age. EL+MV was found to be safe and well-tolerated in this 24-week supplementation study on moderately stressed participants.
